# Genome-wide profiling identifies a subset of methamphetamine (METH)-induced genes associated with METH-induced increased H4K5Ac binding in the rat striatum

**DOI:** 10.1186/1471-2164-14-545

**Published:** 2013-08-12

**Authors:** Jean Lud Cadet, Subramaniam Jayanthi, Michael T McCoy, Bruce Ladenheim, Fabienne Saint-Preux, Elin Lehrmann, Supriyo De, Kevin G Becker, Christie Brannock

**Affiliations:** 1 Molecular Neuropsychiatry Research Branch, DHHS/NIH/NIDA Intramural Research Program, 251 Bayview Boulevard, Baltimore, MD 21224, USA; 2 Gene Expression and Genomics Unit, Intramural Research Program, National Institute on Aging, National Institutes of Health, 251 Bayview Boulevard, Baltimore, MD 21224, USA

**Keywords:** ChIP sequencing, Neural networks, Epigenetics, Histone acetylation, Microarray

## Abstract

**Background:**

METH is an illicit drug of abuse that influences gene expression in the rat striatum. Histone modifications regulate gene transcription.

**Methods:**

We therefore used microarray analysis and genome-scale approaches to examine potential relationships between the effects of METH on gene expression and on DNA binding of histone H4 acetylated at lysine 4 (H4K5Ac) in the rat dorsal striatum of METH-naïve and METH-pretreated rats.

**Results:**

Acute and chronic METH administration caused differential changes in striatal gene expression. METH also increased H4K5Ac binding around the transcriptional start sites (TSSs) of genes in the rat striatum. In order to relate gene expression to histone acetylation, we binned genes of similar expression into groups of 100 genes and proceeded to relate gene expression to H4K5Ac binding. We found a positive correlation between gene expression and H4K5Ac binding in the striatum of control rats. Similar correlations were observed in METH-treated rats. Genes that showed acute METH-induced increased expression in saline-pretreated rats also showed METH-induced increased H4K5Ac binding. The acute METH injection caused similar increases in H4K5Ac binding in METH-pretreated rats, without affecting gene expression to the same degree. Finally, genes that showed METH-induced decreased expression exhibited either decreases or no changes in H4K5Ac binding.

**Conclusion:**

Acute METH injections caused increased gene expression of genes that showed increased H4K5Ac binding near their transcription start sites.

## Background

Methamphetamine (METH) is an illicit psychostimulant that is abused throughout the world. The drug causes behavioral abnormalities that include the development of tolerance and dependence, paranoid states, and psychotic symptoms in human addicts [1,2]. In animals, METH causes behavioral sensitization, is self-administered, and causes structural plasticity in the brain [3-6]. The acute behavioral effects of the drug are mediated by METH-induced increases in the amount of dopamine (DA) release in brain synapses [7,8] and by subsequent stimulation of DA receptors in various brain regions [9]. Activation of these receptors by direct or indirect agonists such as cocaine and amphetamine induces acute changes in the expression of several immediate early genes (IEGs) in the striatum [10,11]. These observations have prompted suggestions that the enduring behavioral and cognitive effects of these psychostimulants might be dependent on transcriptional changes in the rat brain [12,13]. Similarly, acute METH injections cause significant increases in the expression of several IEGs in the rat brain [14] - [17]. These genes include c-fos and Egr1, among others [9,16].

In contrast to the acute METH-induced transcriptional changes, chronic METH administration produces differential changes in IEG responses and blunts the effects of an acute single METH injection on the expression of several IEGs in the striatum [18]. These observations had suggested that chronic METH exposure might alter the molecular machinery that controls the acute transcriptional effects of the drug. These blunting effects might be consequent to alterations in the complex interactions of factors that regulate gene transcription [19,20]. During resting states, DNA is compacted in ways that interfere with the binding of transcription factors whereas DNA becomes more easily accessible during activation of cells by various stimuli [21-23]. DNA is indeed packaged into chromatin whose fundamental subunit, the nucleosome, is made of 4 core histones, histones H2A, H2B, H3, and H4 that form an octomer (2 of each histone) surrounded by 146 bp of DNA [24,25]. Biological processes are regulated, in part, via post-translational modifications of these histones, modifications that include acetylation, methylation, phosphorylation, and ubiquitination [26-30]. Lysine residues of histone tails can be reversibly acetylated and deacetylated by several histone acetyltransferases (HATs) and histone deacetylases (HDACs), respectively [31-33] and these modifications promote alterations in gene expression by enabling or inhibiting recruitment of regulatory factors onto DNA regulatory sequences [31,33,34].

In order to understand the relationship between METH-induced changes in gene expression and histone acetylation on a genome-wide scale, we used two unbiased approaches, namely microarray analyses and chromatin immunoprecipitation (ChIP) followed by massive sequencing [35,36]. In the case of psychostimulants including cocaine, investigators have focused their studies, for the most part, on the effects of illicit drugs on histone H3 modifications [37,38]. However, we chose to investigate METH-induced changes on histone H4 acetylated at lysine residue 5 (H4K5Ac) because a strong link exists between gene activation and acetylation of lysine residues (K5, K8, K12, and K16) of histone H4 [39-42]. We thus used ChIP-Seq to identify sites of binding of H4K5Ac throughout the rat genome. We found that H4K5Ac binding is ubiquitous in the rat dorsal striatum and occurs mainly around transcription start sites (TSSs). There was also a positive correlation between global H4K5Ac binding and striatal gene expression. Moreover, acute METH-induced increases in gene expression were associated with METH-induced increased H4K5Ac binding on genes with increased expression in rats chronically pre-exposed to either saline or METH. Thus, our results document a relationship between H4K5Ac binding and increased gene expression on a global scale.

## Results

### Acute and chronic METH administration causes differential changes in striatal gene expression

We used microarray analysis (RatRef-12 Expression BeadChips arrays, 22, 523 probes, obtained from Illumina Inc., San Diego, CA) to provide a panoramic view of the effects of METH on gene expression in rats chronically exposed to either saline or METH. Using the data obtained from these analyses, we sought to identify molecular and cellular functions of genes with the highest baseline expression in the striatum of control rats. Towards that end, we picked the top 10 percent of genes with the highest expression and ran them through Ingenuity pathway Analysis (IPA). We found that these genes were involved in nucleic acid metabolism (126 genes), post-translational modifications (72 genes), protein folding (26 genes), and cell death and survival (525 genes). They also participate in nervous system development and function (303 genes), mediation of behaviors (166 genes), as well as neurological (447 genes) and psychological (181 genes) diseases. Top canonical pathways include genes involved in mitochondrial functions, EIF2 signaling, protein ubiquitination, mTOR signaling, CDK5 signaling, dopamine-DARPP32 feedback in cAMP signaling, NRF2-mediated oxidative stress response, and synaptic long-term potentiation. The high number of genes involved in these pathways is consistent with the role of dopamine in the striatum [43] and the energy demand and production during brain functions, as well as the role of mitochondrial dysfunction in neurodegenerative disorders [44]. The high expression of these genes in this brain structure supports the notion that the striatum is very sensitive to mitochondrial oxidative dysfunctions [45].

**Figure 1:**
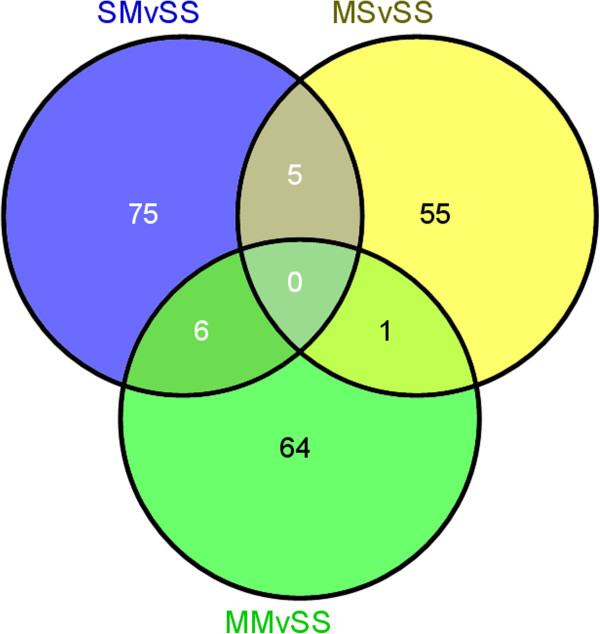
**Microarray analysis identifies selective groups of genes that are differentially expressed after an acute METH injection into METH-naive and METH-pretreated rats.** The Venn diagram shows the overlap of the effects of METH on gene expression. Genes were identified as altered by METH if they showed greater than + 1.7-fold changes (p < 0.01) in mRNA levels in the array experiments. Only 6 genes show overlap between the SM and MM groups. The full list of the METH-regulated genes is given in Additional file 1: Tables S3 and S4.

**Figure 2:**
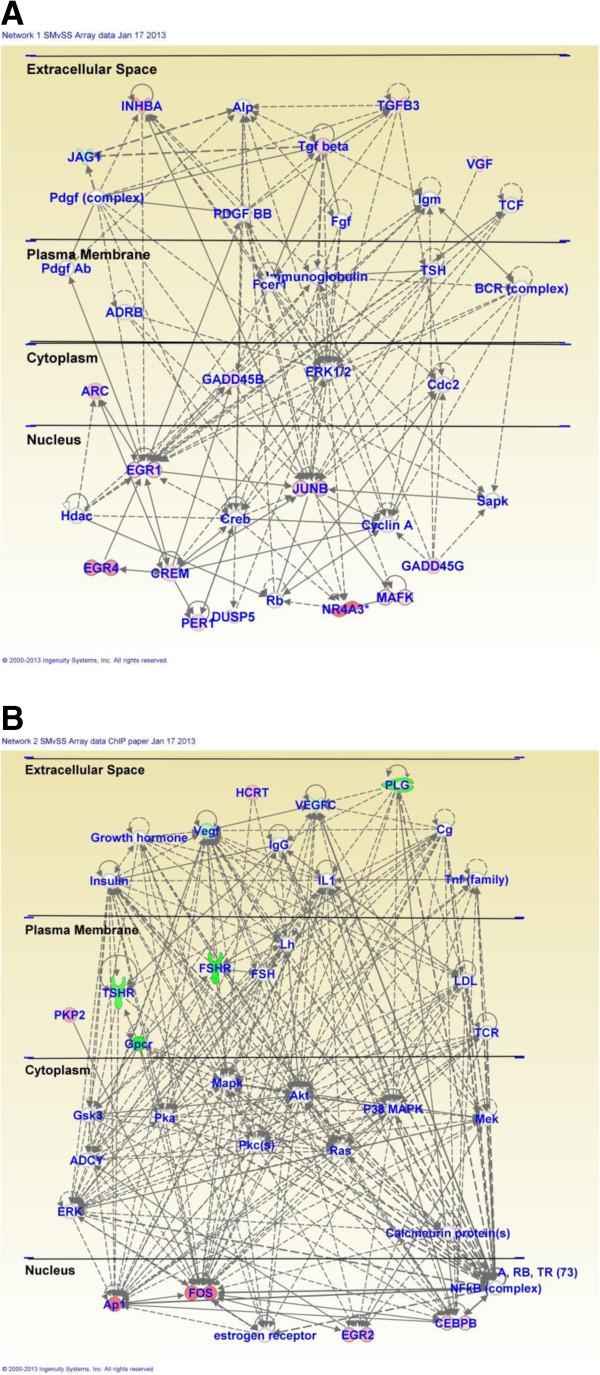
**Pathway analysis of METH-regulated genes in METH-naïve rats.** METH caused changes in the expression of genes involved **(A)** in the control of gene expression and cellular compromise and **(B)** in the regulation of endocrine system development and functions.

Acute injection of METH (5 mg/kg) in METH-naïve rats (SMvSS) caused significant changes in the expression of 86 genes, with 60 being upregulated and 26 downregulated (Figure 1). IPA analysis revealed that these genes are involved in the control of gene expression, participate in cell signaling, regulate cellular growth and proliferation, control organ morphology, and participates in the manifestation of behaviors. Figure 2 shows networks of genes that are involved in the control of gene expression, cellular compromise, and endocrine system development. Upregulated genes found in these networks include several transcription factors, namely Arc, c-fos, Crem, Egr1, Egr2, Egr4, c-fos, junB, Npas4, Nptx2, Nr4a3 (NOR-1) (Figure 2 and Additional file 1: Table S3). The expression of some of these is known to be influenced by illicit drugs, including cocaine [46] and METH [9,14]. Other genes of interests include Dusp14, neurotensin, and orexin-A (hypocretin, HCRT) that are also upregulated (Additional file 1: Table S3). Top canonical pathways that involve these genes include GADD45 signaling, TGFbeta signaling, acute phase response signaling, and NRF2-mediated oxidative stress.

**Figure 3:**
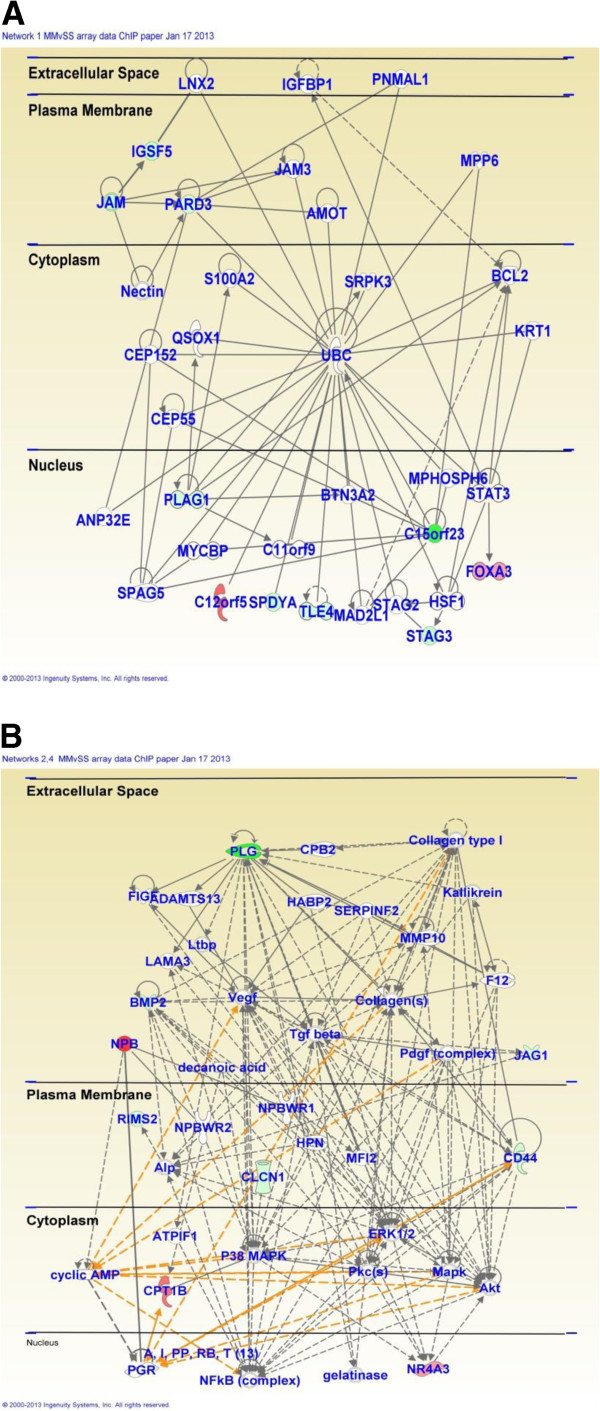
**Pathway analysis of METH-regulated genes in METH-pretreated rats.** METH caused changes in the expression of genes that participate **(A)** in cell cycle and drug metabolism and **(B)** in reproductive system development and function.

**Figure 4:**
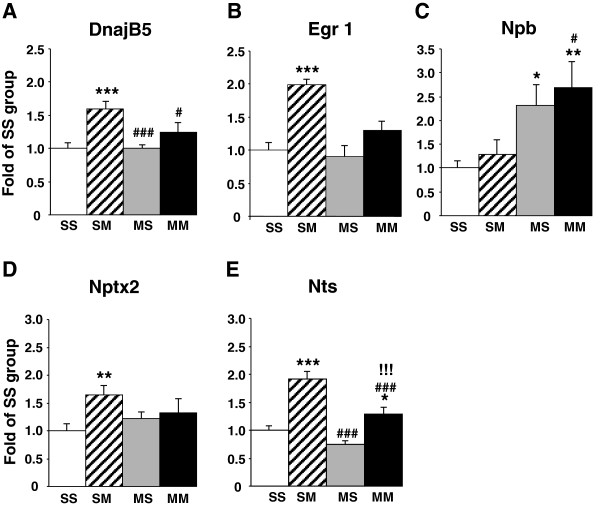
**Quantitative PCR confirmed the METH-induced changes in gene expression.** Acute METH administration increased the expression of **(A)** DnajB5, **(B)** Egr1, **(D)** Nptx2, and **(E)** Nts, but not **(C)** Npb in METH-naïve rats. In contrast, the acute METH injection caused increases in the expression of **(C)** Npb and **(E)** Nts in the METH-pretreated rats, with the effects of acute METH on Nts expression being significantly attenuated in the METH-pretreated rats.

In contrast, acute injection of METH in METH-pretreated rats (MMvSS) caused significant alterations in the expression of 71 genes, with only 18 being upregulated and 53 being downregulated. The list of genes that were affected by the acute METH administration to the chronically METH-treated rats are shown in Additional file 1: Table S4. These genes are involved in cellular development, cell-to-cell signaling and interaction, and carbohydrate metabolism. Top canonical pathways include glioma invasiveness and Rac signaling. The list of genes includes Npb and Nr4a3 that are upregulated and BMP2 that is downregulated by the acute METH injection to rats pre-exposed to the drug. Figure 3 shows networks of genes that are involved in cell cycle, drug metabolism, tissue development, and reproductive system development and function. Figure 4 shows quantitative PCR validation of the METH-induced changes in the expression of some genes of interest in METH-naïve and METH-pretreated rats. These are DnajB5, Egr1, Nptx2, Nts, and Npb.

### Genome-wide analysis of H4K5Ac binding in the rat striatum after METH exposure

**Figure 5:**
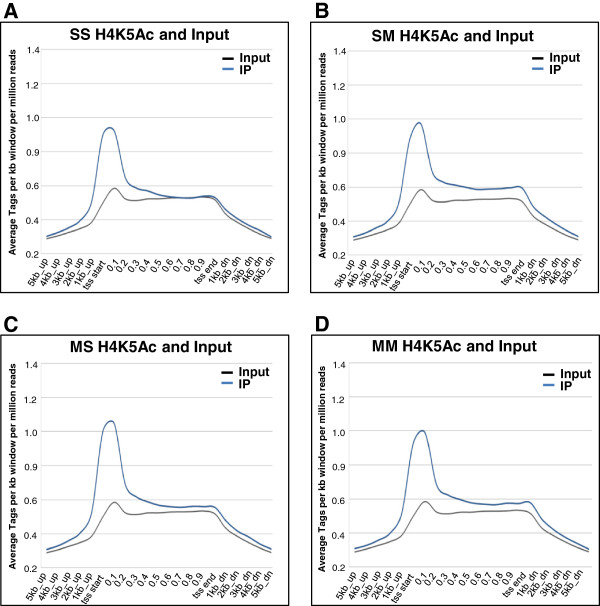
**H4K5Ac binding occurs at the TSSs of genes.** The figure shows the distribution of H4K5Ac binding in **(A)** control rats (SS), **(B)** METH-naïve rats treated with an acute injection of METH (SM), **(C)** rats chronically exposed to METH and then treated acutely with saline (MS), and **(D)** rats chronically exposed to METH and then given an acute injection of METH before being euthanized (MM). The pattern of H4K5Ac binding in the brain was not influenced by neither acute nor chronic METH exposure.

**Figure 6:**
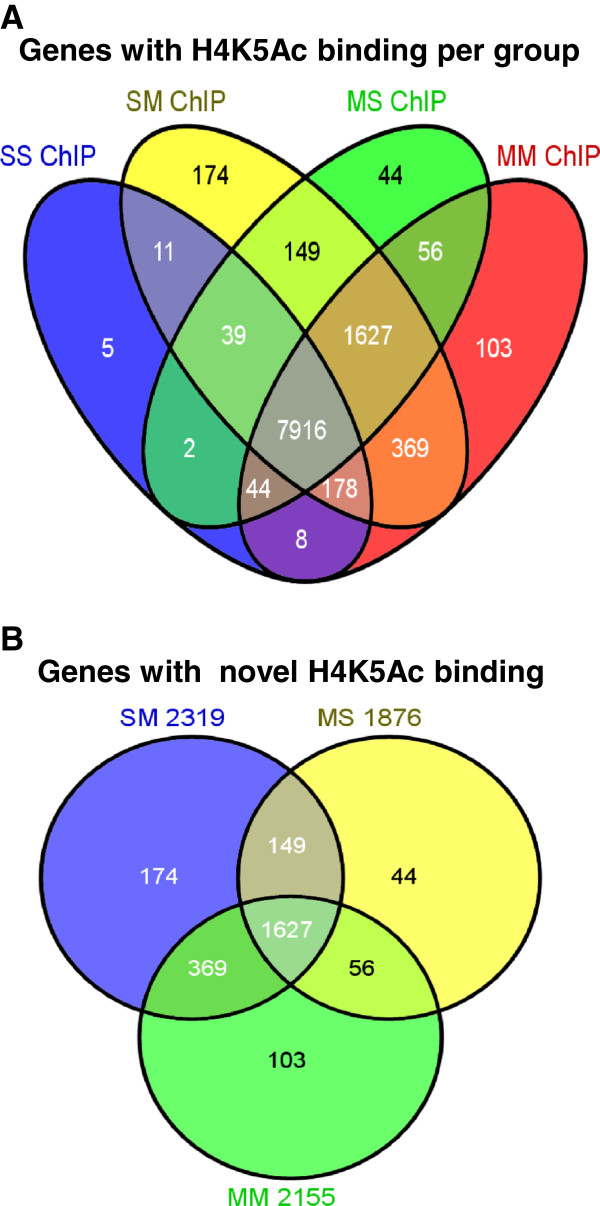
**ChIP-Seq analysis shows that methamphetamine caused large-scale increased H4K5Ac binding in the rat striatum.** The Venn diagram in **(A)** shows the overlap of the effects of acute and chronic METH administration on H4K5Ac binding in the four experimental groups: SS, SM, MS, and MM groups. The Venn diagram in **(B)** shows additional H4K5Ac binding sites in the groups treated with METH in comparison with the control (SS) group.

Although we have consistently shown that acute administration of various doses of METH can cause substantial alterations in gene expression [14-16,47], the epigenetic events involved in these changes have yet to be characterized. Gene expression in the central nervous system is regulated, in part, by epigenetic alterations that include post-translational modifications of histone tails including histone acetylation and methylation [48]. Changes in large-scale DNA binding by modified histones and other proteins, after various manipulations, are now being investigated using ChIP-Seq [35,36,49,50]. We reasoned that a similar approach might help us to identify epigenetic alterations that participate in the acute effects of METH on gene expression in the rat dorsal striatum of METH-naïve and METH-pretreated rats. As shown in Figure 5, genome-wide analysis of H4K5AC binding reveals that H4K5Ac binds around the transcription start sites (TSSs) of genes in the control (Figure 5A), SM (Figure 5B), MS (Figure 5C), and in the MM (Figure 5D) groups. However, there were additional H4K5Ac binding sites in the SM (87,089 H4K5Ac binding sites corresponding to 10,463 annotated genes), MS (50,031 binding sites, 9,877 genes), and the MM (74,856 binding sites, 10,301 genes) groups in comparison to the control animals that showed 22,262 H4K5Ac binding sites corresponding to 8,203 annotated genes in the rat striatum (Figure 6). The majority of genes with H4K5Ac binding in the SS group were also found in the SM, MS, and MM groups (Figure 6). As shown in the figure, 99% of the genes with H4K5Ac binding sites in the control rats (SS) were also found in the METH-naïve rats that received an acute METH injection. Similarly, the majority (97%) of the genes with H4K5Ac binding sites in the control rats were also found in the chronic METH-treated groups, while 99% of the genes in the control group were also found in the MM group. Taken together, these data suggest that both acute and chronic treatment with METH caused the appearance of *de novo* H4K5Ac binding sites in a large number of genes that are expressed in the striatum. Figure 6A also reveals that the vast majority of genes with H4K5Ac binding sites in the groups that had received either acute or chronic METH treatments were co-localized: 9,731 genes in SM and MS, 9,643 genes in MS and MM, 10,090 genes in SM and MM, and 9,543 genes in the 3 METH groups. Figure 6B also shows the majority of METH-induced additional H4K5Ac binding sites were located on genes that were commonly found (1627 annotated genes) in the 3 METH groups. In addition, 1776 genes were common in the SM and MS groups, 1996 genes in the SM and MM groups, and 1683 genes in the MS and MM groups. These results indicate that METH administration exerts consistent effects on H4K5Ac binding in the rodent brain.

Pathway analyses revealed that genes with novel H4K5Ac binding in the SM group are involved in protein synthesis (93 genes), cellular growth and proliferation (539 genes), cell death and survival (582 genes), nervous system development and function (304 genes), behaviors (188 genes), and neurological diseases (358 genes). Top canonical pathways include Ox40 signaling pathway, acute phase response signaling, death receptor signaling, and Huntington’s disease signaling. The genes with novel H4K5Ac binding in the MM group participate in the control of cell death and survival (552 genes), nervous system development and function (264 genes), and neurological diseases (356 genes). Top canonical pathways included OX40 signaling, acute phase response signaling, death receptor signaling, G-protein-coupled receptor signaling, cAMP-mediated signaling, and Huntington Disease signaling. The data on the involvement of AMPK and G-protein receptor signaling are consistent with the known effects of METH on neurotransmitters and their receptors [9].

We next chose the top 10% of genes with highest H4K5Ac binding in the SS, SM, and MM groups for further pathway analyses because we thought that they might potentially play important roles in the functions of the striatum in the absence or presence of METH exposure. IPA revealed that the top 10 percent of the genes with high H4K5Ac binding in the SS group are involved in neurological diseases (61 genes), cancer (58 genes), and developmental disorders (32 genes). Molecular and cellular functions in which they participate include cell cycle regulation (32 genes) and lipid metabolism (10 genes). They are also involved in tissue development (44 genes) and nervous system development and function (41 genes). Top canonical pathways include cAMP-mediated signaling, protein ubiquitination pathway, NRF2-mediated oxidative stress, G-protein-coupled receptor signaling, and tuna splicing. The top 10 percent of genes with high H4K5Ac binding in the SM group are involved in neurological diseases (208 genes) and developmental disorders (91 genes). They also participate in the control of cellular assembly and organization (159 genes), nervous system development and function (198 genes) and behavior (109 genes). Top canonical pathways include protein kinase A signaling, G-protein-coupled receptor signaling, CDK5 signaling, ERK/MAPK signaling, axonal guidance signaling, and Dopamine-DARPP32 feedback in cAMP signaling. Finally, in the MM group, genes in the top 10 percent of high H4K5Ac binding belong to genes that participate in control of gene expression (188 genes), cellular function and maintenance (209 genes), and cell morphology (207). They are also involved in neurological diseases (216 genes), developmental disorders (103 genes), and in nervous system development and function (227 genes). In addition, top canonical pathways in the MM group include molecular mechanisms of cancer, Wnt/beta-catenin signaling, Dopamine-DARPP32 feedback in cAMP signaling, and in G-protein-coupled receptor signaling. Together, these observations are consistent with the idea that acute and chronic METH administration can influence histone acetylation in the brain.

### Global striatal gene expression levels correlate with H4K5Ac binding

**Figure 7:**
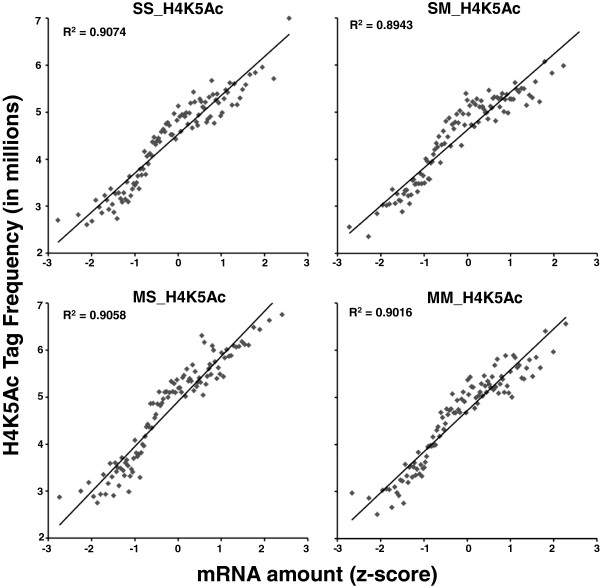
**H4K5Ac binding positively correlates with gene expression in the rat dorsal striatum.** ChIP-Seq and expression data were compared as described previously [51]. In short, genes were sorted based on gene expression values (Z scores) and binned into groups of 100 genes. The average gene expression value for each bin was then calculated. H4K5Ac tags were assigned to the nearest promoter region of genes and normalized to the total tag counts for that sample. The mean tag counts of the above mention bins were also calculated. The averaged binned gene expression values were then graphed against mean tag counts for each bin. The values in the insets represent the regression coefficients (R^2^).

In order to test if H4K5Ac binding correlated with striatal gene expression, we carried out regression analyses and the gene expression and the ChIP-Seq data were compared as described previously [51]. Figure 7 shows that there were positive correlations between the levels of striatal H4K5Ac binding and gene expression in the control (Figure 7A), SM (Figure 7B), MS (Figure 7C), and MM (Figure 7D) groups. These data obtained from the rat brain provide further support for the notion that H4K5 acetylation is an important factor in gene transcription, as reported previously using other models [39-41,52].

### Acute METH-inducible genes are accompanied by METH-induced increased H4K5Ac binding

**Figure 8:**
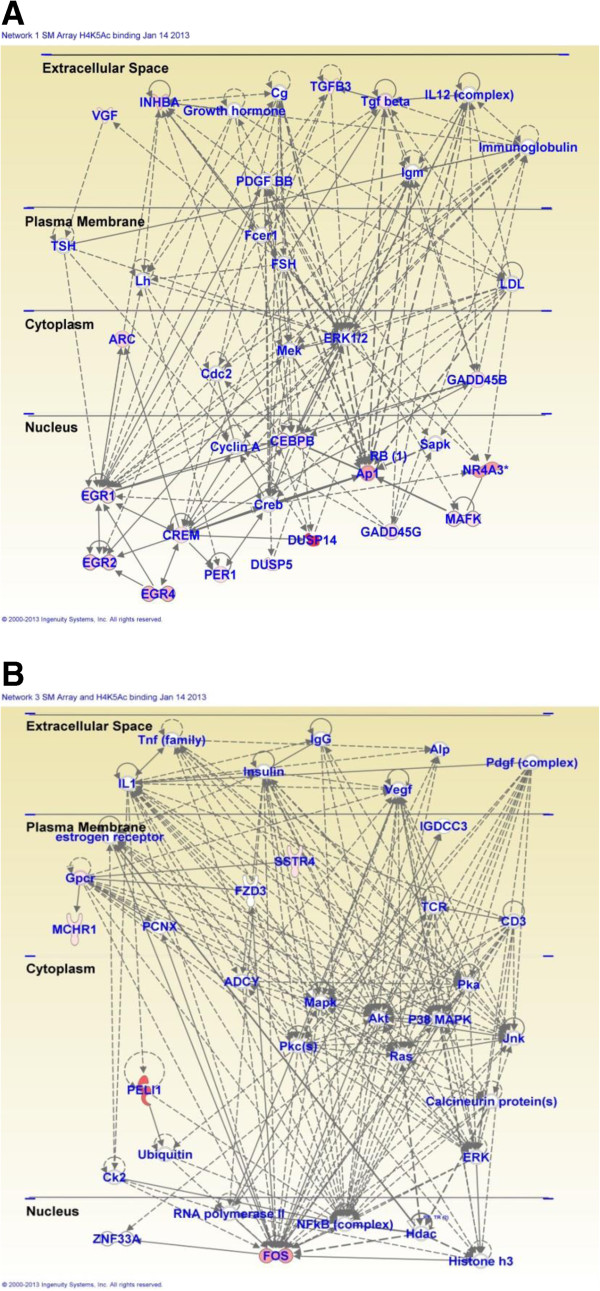
**Acute METH induces increases in gene expression and H4K5ac binding at the TSS of genes involved in embryonic development, behavior, and nervous system development in METH-naïve rats. (A)** IPA of genes involved embryonic development and **(B)** IPA of genes that control behaviors and nervous system development.

As reported above, the acute METH injection caused increased expression of 60 and decreased expression of 26 genes in METH-naïve rats (SM group). We thus wanted to know if METH-induced increased H4K5Ac binding might be related to increased gene expression caused by the drug. Towards that end, we compared H4K5Ac binding between the SS and SM groups among the genes that showed acute changes after the METH injection. We found that 29 of 32 annotated genes present in the array and ChIP-Seq data showed acute METH-induced increased gene expression and increased H4K5Ac binding, while the other 3 genes showed no changes (Table 1). These genes include Arc, c-fos, Egr1, and Crem that are known to be involved in the actions of psychostimulants, including cocaine, in the brain [46,53-55]. Together, these observations support a common role for these genes in drug-induced neuroadaptations in the brain. In contrast, 3 of the 5 down-regulated genes identified on both platforms show no changes while 2 genes showed decreased H4K5Ac binding (Table 1). IPA revealed that the 29 genes with increased expression and H4K5Ac binding are involved in the control of gene expression (16 genes), cellular development (15 genes), cell death and survival (18 genes), and organism development (17 genes). Top canonical pathways include GADD45 signaling, TGF-beta signaling, NRF2-mediated oxidative stress response, and protein kinaseA signaling. Figure 8 shows networks of genes that are involved in embryonic development and cellular compromise (Figure 8A), and in the control of nervous system development and behavior (Figure 8B).

**Table 1:** Effects of acute METH injection on gene expression and H4K5Ac binding in METH-naïve rats

**Symbol**	**Definition**	**FC**	**H4K5Ac binding**
Dusp14	Dual specificity phosphatase 14	13.17	4.35
Peli1	Pellino homolog 1 (Drosophila)	10.64	4.97
Npas4	Neuronal PAS domain protein 4	9.04	3.97
Prodh2	Proline dehydrogenase (oxidase) 2	6.95	4.24
Olr464	Olfactory receptor 464	6.51	3.71
Nr4a3	Nuclear receptor subfamily 4, group A, member 3	6.43	6.01
Fos	FBJ murine osteosarcoma viral oncogene homolog	6.18	4.77
Nr4a3	Nuclear receptor subfamily 4, group A, member 3	4.71	4.39
Egr4	Early growth response 4	4.62	*
Fzd7	Frizzled homolog 7 (Drosophila)	4.22	*
Pkp2	Plakophilin 2	3.79	NC
Nptx2	Neuronal pentraxin II	3.77	2.44
Inhba	Inhibin beta-A	3.71	*
Dhrs9	Dehydrogenase/reductase (SDR family) member 9	3.54	NC
Hcrt	Hypocretin	3.08	3.07
Egr2	Early growth response 2	3.08	3.49
Arc	Activity regulated cytoskeletal-associated protein	2.88	NC
Junb	Jun-B oncogene	2.36	*
Taar8c	Trace-amine-associated receptor 8c	2.33	*
Nts	Neurotensin	2.32	2.67
Gadd45g	Growth arrest and DNA-damage-inducible 45 gamma	2.21	5.30
Dnajb5	DnaJ (Hsp40) homolog, subfamily B, member 5	2.03	2.28
Cebpb	CCAAT/enhancer binding protein (C/EBP)	1.99	2.75
Tgfb3	Transforming growth factor, beta 3	1.98	6.55
Egr1	Early growth response 1	1.89	*
Baz1a	Bromodomain adjacent to zinc finger domain, 1A	1.89	3.13
Dusp5	Dual specificity phosphatase 5	1.84	*
Porf1	Preoptic regulatory factor 1	1.83	2.48
Per1	Period homolog 1 (Drosophila)	1.83	3.48
Ptpdc1	Protein tyrosine phosphatase domain containing 1.	1.82	4.24
Mchr1	Melanin-concentrating hormone receptor 1	1.81	3.79
Gpd1	Glycerol-3-phosphate dehydrogenase 1 (soluble).	1.80	3.70
Abcc10	ATP-binding cassette, sub-family C (CFTR/MRP)	1.78	5.25
Crem	cAMP responsive element modulator	1.77	3.34
Mafk	v-maf musculoaponeurotic fibrosarcoma oncogene family	1.77	3.21
Vgf	VGF nerve growth factor inducible	1.76	3.67
Sstr4	Somatostatin receptor 4	1.75	3.86
Gadd45b	Growth arrest and DNA-damage-inducible 45 beta	1.72	3.26
Snf1lk	SNF1-like kinase	1.72	3.07
Asb1	Ankyrin repeat and SOCS box-containing protein 1	1.71	*
Pip3ap	Phosphatidylinositol-3-phosphatase associated protein	1.70	*
Stag3	Stromal antigen 3	−1.85	*
Gpr149	G protein-coupled receptor 149	−1.85	NC
Vegfc	Vascular endothelial growth factor C	−1.90	−1.49
Jag1	Jagged 1 (Jag1), mRNA.	−1.94	−3.50
Olr990	Olfactory receptor 990 (Olr990), mRNA.	−4.34	*
Amy1	Amylase 1, salivary (Amy1), mRNA.	−4.54	*
Tshr	Thyroid stimulating hormone receptor (Tshr), mRNA.	−4.85	*
Dao1	D-amino acid oxidase 1 (Dao1), mRNA.	−4.94	NC
Plg	Plasminogen (Plg), mRNA.	−5.37	*
Olr1463	Olfactory receptor 1463 (Olr1463), mRNA.	−5.61	NC
Olr462	Olfactory receptor 462 (Olr462), mRNA.	−5.87	*
Fshr	Follicle stimulating hormone receptor (Fshr), mRNA.	−6.06	*

Gene expression was measured by microarray analysis. Genes were identified as showing changes in expression if they showed greater than ±1.7-fold changes (p < 0.01) in comparison to the control group. H4K5Ac binding was measured by ChIP-Seq assays. H4K5Ac binding was calculated by comparing the control and METH-treated groups, after corrections for DNA inputs. *Abbreviations*: *FC* fold changes, *NC* no changes in H4K5Ac binding, *, no H4K5Ac binding sites observed.

**Figure 9:**
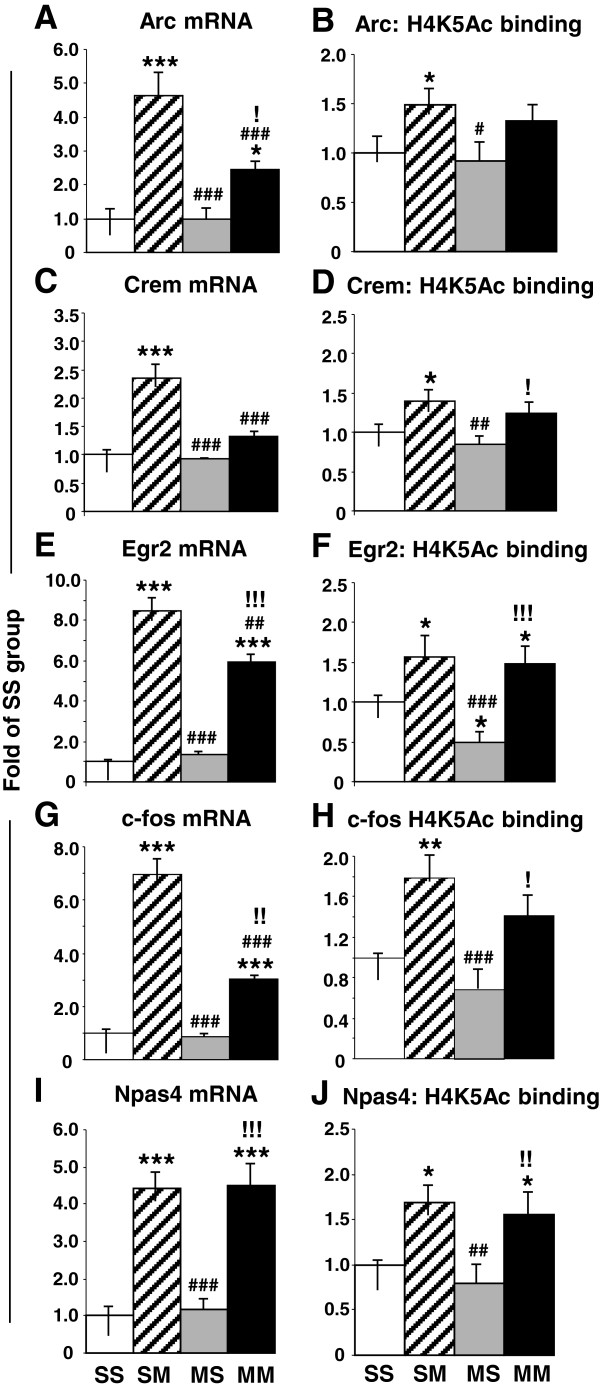
**Real-time PCR validation of the changes of expression and in H4K5Ac binding observed in genes of interest.** Quantitative PCR shows that acute METH induced significant increases in the expression of **(A)** Arc, **(C)** Crem, **(E)** Egr2, **(G)** c-fos, and **(I)** Npas4 in saline pre-treated rats. Acute METH also caused smaller increases in **(A)** Arc, **(E)** Egr2, **(G)** c-fos, expression in METH-pretreated rats. In contrast, acute METH produced similar increases in **(I)** Npas4 expression in both saline- and METH-pretreated animals. The figure also shows the results of ChIP-PCR that revealed acute METH-induced increases in H4K5ac binding around the TSSs of **(B)** Arc, **(D)** Crem, **(F)** Egr2, **(H)** c-fos, and **(J)** Npas4 in saline-pretreated rats. METH-induced increased H4K5Ac binding in the METH-pretreated rats was observed only around the TSSs of **(F)** Egr2 and **(J)** Npas4. The data represent means ± SEM of 5–8 animals per group. Statistical analyses are described in the text. *, **, *** = p < 0.05, 0.001, 0.001, respectively, in comparison to the SS group; #, ##, ### = p < 0.05, 0.01, 0.001, respectively in comparison to the SM group; !, !!, !!! = p < 0.05, 0.01, 0.001, respectively in comparison to the MS group.

Quantitative PCR confirmed the changes in gene expression after acute administration of the drug. Acute METH causes significant increases in the expression of Arc (3-fold, p < 0.0001) (Figure 9A), Crem (2.1-fold, p < 0.0001) (Figure 9C), Egr2 (8.5-fold, p < 0.0001) (Figure 9E), c-fos (7-fold, p < 0.0001) (Figure 9G), and Npas4 (4.4-fold, p <0.0001) (Figure 9I) in saline pre-treated rats. In METH-pretreated rats, acute METH also caused smaller increases in Arc (1.8-fold, p = 0.034) (Figure 9A), Egr2 (5.9-fold, p < 0.001) (Figure 9E), c-fos, (3-fold, p = 0.0027) (Figure 9G) but not in Crem (Figure 9C). In contrast, Npas4 showed similar increases in saline- and METH-pretreated (4.5-fold, p < 0.0001) (compare SM to MM groups in Figure 9I) mRNA levels after the acute METH injection. Interestingly, we found significant increases in Arc, Egr2, and c-fos mRNA by quantitative PCR but not on the microarray analyses. These observations are probably due to the stringent criteria that we used in the microarray and the more quantitative nature of the PCR analyses.

ChIP-PCR confirmed the ChIP-Seq data and showed that acute METH caused significant increases in H4K5ac binding around the TSSs of Arc (1.5-fold, p = 0.029) (Figure 9B), Crem (1.4-fold, p = 0.023) (Figure 9D), Egr2 (1.6-fold, p = 0.015) (Figure 9F), c-fos (1.8-fold, p = 0.0021) (Figure 9H), and Npas4 (1.7-fold, p = 0.012) (Figure 9J) in saline-pretreated rats. In contrast, there was METH-induced increased H4K5Ac binding in the METH-pretreated rats only around the TSSs of Egr2 (1.5-fold, p = 0.040) (Figure 9F) and Npas4 (1-6-fold, p = 0.0013) (Figure 9J).

**Figure 10:**
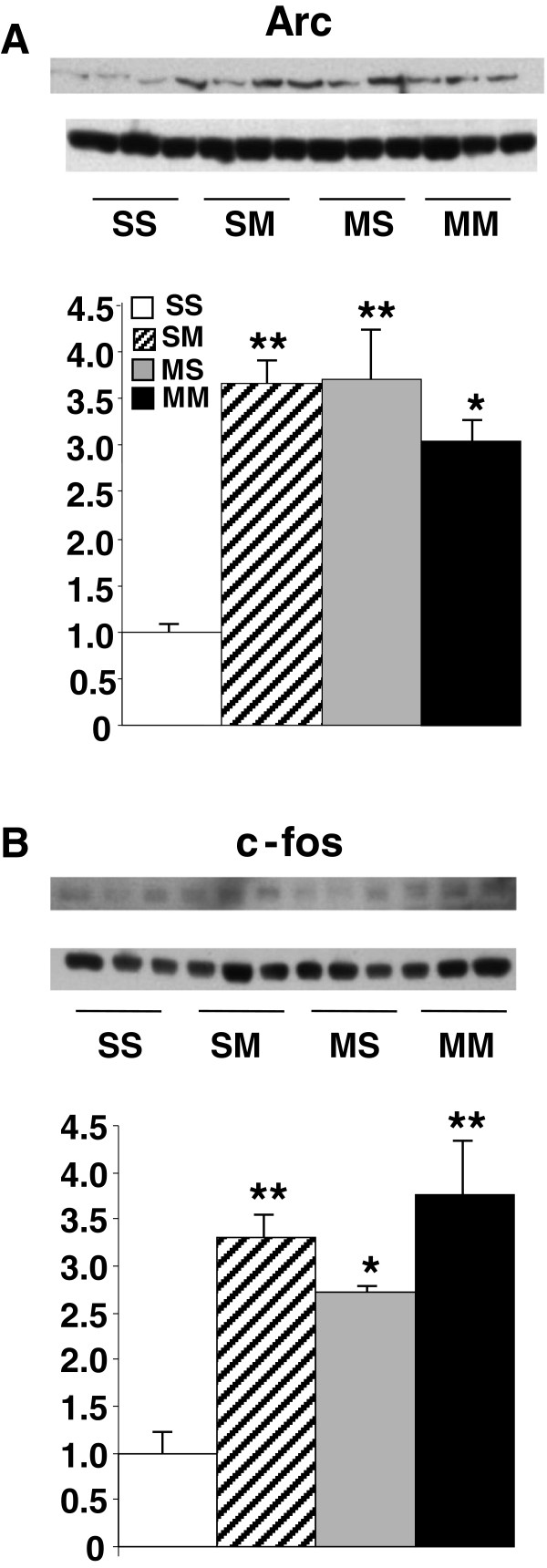
**METH administration caused increased Arc (A) and c-fos (B) protein expression in the rat striatum.** The data represent means ±SEM of 5–8 animals per group. Statistical analyses are described in the text. *, **, = p < 0.005, 0.001, respectively, in comparison to the SS group.

We also measured the protein expression of Arc and c-fos (Figure 10). Acute METH caused significant increases in Arc (3.67-fold, p = 0.0003) (Figure 10A) and c-fos (3.31-fold, p = 0.0008) (Figure 10B) expression in saline-pretreated rats. Repeated exposure to METH also caused increased Arc (3.7-fold, p = 0.0003) and c-fos (2.72-fold, p = 0.0049) protein expression. Moreover, there were significant increases in Arc (3.04-fold, p = 0.0017) and c-fos (3.76-fold, p = 0.0003) protein expression in the MM group.

**Figure 11:**
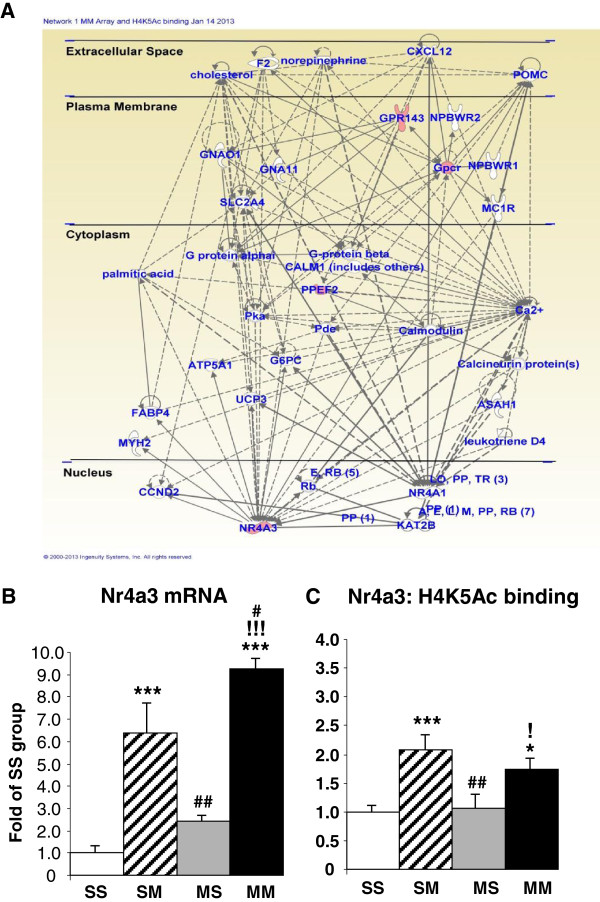
**METH produces increased expression and increased H4K5Ac binding in a small number of genes in METH-pretreated rats. (A)**. METH increased gene expression and H4K5Ac binding in TSSs of genes involved in molecular transport and in carbohydrate and lipid metabolism. Quantitative PCR was used to validate the results of the microarray **(B)** and ChIP-Seq **(C)** analyses. Statistical analyses are described in the text. *, **, *** = p < 0.05, 0.001, 0.001, respectively, in comparison to the SS group; #, ##, ### = p < 0.05, 0.01, 0.001, respectively in comparison to the SM group; !, !!, !!! = p < 0.05, 0.01, 0.001, respectively in comparison to the MS group.

As reported above, the acute administration of METH to METH-pretreated rats caused changes in the expression of 71 genes, with most genes being downregulated (Figure 1). Table 2 shows that 4 of the 5 upregulated genes identified on both platforms showed increased H4K5Ac binding whereas one gene showed no changes in binding. These genes included Npb and Nr4a3. IPA shows that they are involved in cellular development (Nr4a3), cell morphology (Gpr143), tissue development (Nr4a3 and Gpr143), hereditary disorders (Gpr143 and Ppef2), and reproductive system development and function (Npb). Figure 11A shows that these genes are involved in networks that participate in carbohydrate and lipid metabolism. In contrast, 7 of the 14 downregulated annotated genes identified on both platforms showed no changes while the other 7 showed decreased H4K5Ac binding (Table 2). We also used quantitative PCR to confirm the METH-induced increases in Nr4a3 mRNA in the SM (6.4-fold, p < 0.0001) and MM (9.3-fold, p < 0.0001) groups (Figure 11B). ChIP-PCR also confirmed the changes in H4K5Ac binding in the SM (2-fold, p = 0.0004) and MM (1.7-fold, p = 0.011) groups (Figure 11C).

**Table 2:** Effects of acute METH injection on gene expression and H4K5Ac binding in METH-pretreated rats

**Symbol**	**Definition**	**FC**	**Binding**
Olr577	Olfactory receptor 577	6.39	*
Npb	Neuropeptide B	5.70	4.22
Plagl2	Pleiomorphic adenoma gene-like 2	4.67	*
Cpt1b	Carnitine palmitoyltransferase 1b, muscle	3.91	*
Ppef2	Protein phosphatase, EF hand calcium-binding domain	3.07	2.95
Gpr143	G protein-coupled receptor 143 (Gpr143), mRNA.	2.88	4.88
Nr4a3	Nuclear receptor subfamily 4, group A, member 3	2.79	4.22
Foxa3	Forkhead box A3	2.60	NC
Olr734	Olfactory receptor 734	2.28	*
Olfml2a	Olfactomedin-like 2A	−1.71	NC
Zfp533	Zinc finger protein 533	−1.73	*
Pard3	Par-3 (partitioning defective 3) homolog (C. elegans)	−1.74	−3.94
Tle4	Transducin-like enhancer of split 4, homolog of Drosophila E [20]	−1.75	−4.22
Bcar3	Breast cancer anti-estrogen resistance 3	−1.76	−2.94
Plag1	Pleiomorphic adenoma gene 1	−1.76	*
Rcn1	Reticulocalbin 1	−1.81	*
Gpcr12	G-protein coupled receptor 12	−1.81	NC
Arl6	ADP-ribosylation factor-like 6	−1.87	NC
Prkcm	Protein kinase C, mu	−1.88	*
Clcn1	Chloride channel 1	−1.90	*
Jag1	Jagged 1	−1.94	−3.50
Fbxo15	F-box protein 15	−1.96	*
Bmp2	Bone morphogenetic protein 2	−1.99	−3.14
Cd44	CD44 antigen	−2.01	*
Rims2	Regulating synaptic membrane exocytosis 2	−2.13	−3.50
Gpr149	G protein-coupled receptor 149	−2.14	−3.23
Spdya	Speedy homolog A (Drosophila)	−2.35	NC
Plin	Perilipin	−2.47	*
Stag3	Stromal antigen 3	−2.51	*
Clca3	Chloride channel calcium activated 3	−3.77	*
Cldn22	Claudin 22	−3.89	NC
Nek1	NIMA (never in mitosis gene a)-related expressed kinase 1	−4.36	NC
Olr990	Olfactory receptor 990	−4.50	*
Ctcfl	CCCTC-binding factor (zinc finger protein)-like	−5.45	*
Sdfr2	Stromal cell derived factor receptor 2	−6.02	*
Traf4af1	TRAF4 associated factor 1	−8.83	NC
Plg	Plasminogen	−9.93	*

Genes were identified and calculations were performed as described in Table 1. *Abbreviations*: *FC* fold changes, *NC* no changes in H4K5Ac binding, *, no H4K5Ac binding sites observed.

## Discussion and conclusion

Our study provides, for the first time, a comprehensive map of acetylated H5K5Ac binding throughout the rat genome and documents the presence of thousands of these sites in genes expressed in the rat striatum. We also show that H4K5Ac binding occurs around TSSs and that the pattern of binding is not affected by METH treatments. In addition, both acute and chronic METH administration caused significant changes in H4K5Ac binding, with additional binding sites being observed in more genes after the acute METH injections. Moreover, levels of gene expression correlated with genome-wide H4K5Ac binding in the striatum. The microarray analysis further revealed that acute METH also caused increased expression of 60 of 86 genes in saline-pretreated rats whereas there was mostly decreased gene expression (53 of 71 genes) after an acute METH injection to METH-pretreated rats. Important, the vast majority of genes with increased expression also experienced increased H4K5Ac binding while the genes with decreased expression showed either decreases or no changes in H4K5Ac binding. The findings that METH-induced increased H4K5Ac binding is associated with increased expression of a set of genes after the acute METH injection in METH-naïve rats is consistent with the report that all-trans-retinoic acid caused increased histone H4 acetylation and increased gene expression during leukemic cell differentiation [56]. Our data are also consistent with the observation that deletion of the histone deacetylase, RPD3, produced increased H4K5Ac binding at promoters of several genes in Saccharomyces cerevisiae [41]. Nevertheless, the relationship of METH-induced increased H4K5Ac binding to increase gene expression appears to be somewhat complex. For example, METH-induced increased H4K5Ac binding in the SM or MM groups did not necessarily translate into significant METH-induced changes in gene expression as measured by the microarray analysis. This conclusion is consistent with those of other investigators who have reported that individual activators can cause differential patterns of histone acetylation, with some causing increased H4 acetylation but others causing variable effects on H4 acetylation and gene expression [57]. Together, these results suggest that, under the chronic METH condition, METH-induced increased H4K5Ac binding is not sufficient to cause METH-induced increased expression of the majority of genes in the dorsal striatum. These data implicate the existence of other epigenetic factors that might serve to regulate, in conjunction with H4K5Ac binding, the expression of genes that show substantial changes in H4K5Ac binding. This discussion provides a partial explanation for our observation that the acute METH injection caused mostly downregulation of gene expression in the METH-pretreated rats. When taken together with the observations of the existence of epigenetic ensembles that control gene expression in human cells [50,58], our results suggest that combinatorial epigenetic influences [21] might also be responsible for the acute transcriptional changes observed after an acute METH injection to METH-naïve or METH-pretreated rats, with H4K5Ac binding playing a contributory role.

We also used qRT-PCR and ChIP-PCR in order to confirm some of the changes observed using the two discovery platforms. We picked Arc, Crem, Egr2, and Nr4a3 because they are implicated in synaptic plasticity [59-62]. Crem mRNA expression was increased in comparison to the control group only after the acute METH injection to METH-naive rats. H4K5Ac binding around Crem TSS was also increased after the acute METH administration in METH-naïve rats but not in METH-pretreated rats. These observations suggest that chronic METH might have caused additional epigenetic modifications that had rendered Crem expression refractory to the acute effects of the drug. These observations are somewhat dissimilar to our observations of the effects of METH on Egr2 expression. Specifically, the acute METH injection caused substantial increases in Egr2 mRNA in saline-pretreated rats. In contrast, there was attenuation of the acute METH-induced effects on Egr2 expression in the METH-pretreated rats. This attenuation occurred in spite of the fact that acute METH caused increased H4K5Ac binding in both METH-naive and METH-pretreated rats. Egr2 is a member of the Kruppel-like zinc finger transcription factors that include Egr1, Egr3 and Egr4 [62,63]. The Egrs are activated by neuronal activity [62,63] and by METH [14,15]. Egr2 mediates stabilization and maintenance of long-term potentiation (LTP) [64] and regulates attentional processes [65]. Although the role of Egr2 induced by small METH doses is not clear, high METH doses have been shown to cause Egr-dependent activation of Fas ligand (FasL)-mediated neuronal apoptosis [15]. Importantly, the observations that chronic METH pretreatment blunted the acute effects of METH on Crem and Egr2 expression are consistent with data from other investigators who had reported that the acute effects of psychostimulants on IEG expression were blunted in animals previously exposed to either cocaine [46]or the amphetamines [18,66,67]. Altogether, the observation of chronic drug administration-induced blunting effects of the acute transcriptional consequences of psychostimulants suggests that these phenomena might participate in molecular events responsible for drug-induced tolerance [68]. They might also explain, in part, the need for repeated drug-seeking and drug-taking behaviors that are sine qua non of drug addiction [69,70].

It is also of interest to discuss the effects of acute and chronic effects of METH on Nr4a3 expression in contrast to the observations with Crem and Egr2 discussed above. Nr4a3 is a member of Nr4a1/Nur77 family of transcription factors (Nr4a1/Nur77/NGFIB, Nr4a2/Nurr1 and Nr4a3/Nor-1) that belong the superfamily of steroid nuclear hormone receptor superfamily [71,72]. They participate in a number of biological functions including cellular proliferation, differentiation, and apoptosis [71,72]. Nr4a3 also regulates axonal guidance and pyramidal cell survival in the hippocampus [73]. As shown above, PCR assays confirmed both the METH-induced changes in Nr4a3 gene expression and in H4K5Ac binding that were identified by the microarray and ChIP-Seq experiments, respectively. Importantly, we found that chronic METH did not produce blunting of the acute METH-induced increased in Nr4a3 expression in the METH-pretreated rats in contrast to the observations for Crem and Egr2 mRNA expression discussed above. When taken together, our observations hint to a role of these genes as important yet differential regulators of molecular events that are consequent to repeated METH exposure.

In addition to the IEGs, acute METH was found to increase neurotensin mRNA levels and H4K5Ac binding around the Nts TSS in the striatum. We also found that the acute effects of METH on Nts expression were somewhat attenuated in METH-pretreated rats. This is of interest because acute METH administration is known to cause increased neurotensin mRNA and protein expression in the rat striatum [74-76]. Similar increases are also observed in animals trained to self-administer the drug [6,77,78]. Our observations thus add to the literature that indicates that METH influences this neuropeptidergic system in the rat brain. It is important to note that a recent study had reported that neurotensin levels are significantly downregulated in the striatum of rats that had undergone extinction training after METH self-administration [79], suggesting differential responses in neurotensin expression after acute METH and during drug withdrawal. In any case, when taken together, these observations suggest that neurotensin might play an important role in the acute behavioral responses to the drug and/or in the maintenance of METH self-administration. The demonstration that METH also increased H4K5Ac binding at the Nts gene promoter provides a partial explanation for the acute effects of the drug on neurotensin expression in the striatum.

Another peptide of interest is neuropeptide B (NPB) that also shows increased mRNA expression after chronic METH treatments. NPB, a neuropeptide of 29 aa residues, was identified as an endogenous ligand for the G protein-coupled receptor, GPR7, whose stimulation causes decreased intracellular cAMP production [80,81]. The NPB transcript is widely distributed in the brain [81,82]. NPB has been implicated in the regulation of pain sensation, endocrine function, as well as feeding behaviors [81,83-86]. For example, intracerebral NPB injection decreases feeding behaviors [81] whereas NPB-knockout mice are obese [85]. These observations are compatible with the known anorectic effects of the amphetamine analogs including METH [87,88] and suggest that NPB might play a role in METH-induced chronic anorexia. The veracity of this argument will need to be tested experimentally. The role of NPB in other behavioral aspects of METH needs also to be considered. In any case, the present observations add to the growing literature that METH can substantially influence the expression of various neuropeptides and implicate these substances in the acute and long-term neuroplastic effects of the drug.

It is also of interest to discuss some of the METH-induced networks that were identified by pathway analysis. The IPA showed that injections of METH induced the expression of genes that are involved in the development of diverse systems. These genes include Egr1, Egr2, c-fos, Nr4a3, and Vgf (Figures 2, 3 and 8). The METH-induced increased expression of the developmental gene, foxa3 (Figure 3) which is a member of the family of forkhead winged transcription factors [89,90], is of interest because, together with the changes in other transcription factors, these observations support the notion that amphetamine and its analogs might recapitulate developmental programs in adult animals [91]. This idea was initially based on the findings of Webb et al. (2009) [92] who had reported that, in zebrafish, amphetamine induced a set of genes enriched with transcription factors that are known to participate in developmental processes. Our observations are also consistent with the idea that drug addiction is dependent on altered synaptic plasticity [93-96] that are regulated, in part, by developmental factors in adult animals [91,97].

In summary, our study has provided a detailed description of the acute and chronic effects of METH on H4K5Ac binding and gene expression in the brain. We found that acute METH-induced increases in H4K5Ac binding were, in part, responsible for a subset of METH-upregulated genes in METH-naïve and METH-pretreated rats. However, given the appearance of many novel H4K5Ac binding sites in the striatum after both acute and chronic METH administration, the observations of METH-induced changes in the expression of only a few genes suggest that the presence of METH-induced novel H4K5Ac binding sites might be necessary but not sufficient to induce transcriptional changes in gene expression. Moreover, because the acute METH injection caused, for the most part, decreased mRNA levels in METH-pre-exposed rats, the possibility exists that repeated METH exposure might have triggered epigenetic modifications which had negatively impacted the expression of METH-responsive genes. This idea is consistent with the combinatorial nature of epigenetic events that control inducible gene expression [21,50]. Finally, given the adverse neuropsychiatric and psychosocial consequences of METH addiction, similar studies are necessary to help to identify specific long-lasting epigenetic effects of repeated METH exposure. The elucidation of these molecular alterations might help to develop alternative pharmacological approaches for the treatment of this common, yet complex, psychiatric disorder.

## Methods

### Animals

Male Sprague–Dawley rats (Charles Rivers Laboratories, Raleigh, NC), weighing 330–370 g in the beginning of the experiment were used in the present study. Animals were housed in a humidity- and temperature-controlled room and were given free access to food and water. All animal procedures were performed according to the National Institutes of Health Guide for the Care and Use of Laboratory Animals and were approved by the National Institute of Drug Abuse-/ Intramural Research Program (IRP) Animal Care and Use Committee (NIDA/IRP-ACUC).

### Drug treatment and tissue collection

Following habituation, rats were injected intraperitoneally with either (±) METH-hydrochloride (NIDA, Baltimore, MD) or an equivalent volume of 0.9% saline over a period of two weeks as described in Additional file 1: Table S1. The saline- or METH-pretreated animals received a single injection of saline or METH (5 mg/kg × 1) at 16–18 hrs after the last saline or METH pretreatment injection. This dose of METH does not cause any neurotoxic effects, as much larger doses are required for pathological changes to develop in the rodent brain [47]. The four groups of animals were: saline/saline (SS), saline/METH (SM), METH/saline (MS), and METH/METH (MM). The animals were euthanized by decapitation 2 hrs later and their brains were quickly removed. Striatal tissues from one side were dissected on ice, snap frozen on dry ice, and stored at −80°C until used in microarray and quantitative PCR experiments whereas the other side was processed for ChIP experiments detailed below.

### RNA extraction

Total RNA was isolated using Qiagen RNeasy Mini kit (Qiagen, Valencia, CA) according to the manufacturer’s instructions. RNA integrity was assessed using an Agilent 2100 Bioanalyzer (Agilent, Palo Alto, CA) and showed no degradation. The RNA extracted from the striatum was used to measure gene expression by microarray analysis and quantitative PCR was used to confirm the expression of some genes of interest.

### Microarray analysis

Microarray hybridization was carried out using RatRef-12 Expression BeadChips arrays (22, 523 probes) (Illumina Inc., San Diego, CA) essentially as previously described by us [16]. Raw data were imported into GeneSpring and normalized using global normalization. The normalized data were used to identify changes in gene expression after the various patterns of METH injections as described above. A gene was identified as significantly affected if it showed increased or decreased expression according to an arbitrary cut-off of 1.7-fold change at p<0.01, according to the GeneSpring statistical package. Similar criteria have been successfully used in our previous microarray studies [15,16]. Network analyses were performed using the Ingenuity Pathway Analysis (IPA) software (Ingenuity Systems, Redwood City, CA). The IPA software allows for the identification of networks, canonical pathways, and biological functions that are affected by the drug. We also used the IPA software to graphically show the cellular location of genes significantly affected by METH.

### Quantification of mRNA by quantitative real-time PCR

Total RNA was obtained individually from 6–8 rats per group and was reverse-transcribed with oligo dT primers and RT for PCR kit (Clontech, Palo Alto, CA). PCR experiments were done using the Chroma4 RT-PCR Detection System (BioRad Hercules, CA USA) and iQ SYBR Green Supermix (BioRad) according to the manufacturer’s protocol. Sequences for gene-specific primers corresponding to PCR targets were obtained using LightCycler Probe Design software (Roche). The primers were synthesized and HPLC-purified at the Synthesis and Sequencing Facility of Johns Hopkins University (Baltimore, MD). The sequences for the IEG primers have been previously published [15,97]. Additional file 1: Table S2 shows the sequences of the primers. Quantitative PCR values were normalized using OAZ1 (ornithine decarboxylase antizyme 1) based on a previous paper [98]. The results are reported as relative changes calculated as the ratios of normalized gene expression data of each group compared to the SS group. The list of primers is noted in Additional file 1: Table S2.

### ChIP-Seq and ChIP-PCR

Striatal tissues were processedfor ChIP-Seq and ChIP-PCR. Briefly, brain tissues were minced to ~1 mm-sized pieces, and immediatelycross-linked in 1% formaldehyde for 15 min at room temperature. The tissues were washed four times in cold PBS containing the proteinase inhibitors in the Roche protease inhibitor cocktail tablet (Roche Diagnostics) and 1 mMPMSF (Sigma). Tissues were rapidly frozen on dry ice. The fixed tissues were resuspendedin SDS lysis buffer (EMD Millipore Corp) containing the Roche protease inhibitor cocktail and 1 mM PMSF and each sample was transferred to TPX plastic tube (Diagenode Inc., Denville, NJ) and sonicated ^~^15 cycles of 30 sec Time ON and 30 sec Time OFF using a Bioruptor (Diagenode Inc.). Fragmentation was checked by gel analysis to confirm sheared ranges of 300-600 bp. Dynabeads (Life Technologies, Grand Island, NY) were incubated with 5 μg of a specific antibody directed against H4K5Ac for ChIP-Seq. Similarly, samples from another group of animals were incubated with the same antibody to confirm some of the ChIP-Seq data using ChIP-PCR. Sequences for the ChIP-PCR are shown in Additional file 1: Table S2.

For DNA sequencing, adapters were ligated to the precipitated DNA fragments or the input DNA to construct a sequencing library according to the manufacturer’s protocol (Illumina, San Diego, CA). Sequencing images generated were analyzed with the Firecrest program followed by base calling using the Bustard program. The first 41 bases were aligned to the rat reference genome using the Gerald program. Firecrest, Bustard and Gerald are part of the Illumina Analysis Pipeline package. H4K5Ac binding was identified by ChIP-Seq and was calculated by comparing the control and METH-treated groups after corrections for DNA inputs. The microarray and ChIP-Seq data have been deposited in NCBI under GEO accession number GSE42776. ChIP-Seq and expression data were compared as described previously [51]. In short, genes were sorted based on gene expression values (Z scores) and binned into groups of 100 genes. The average gene expression value for each bin was then calculated. H4K5Ac tags were assigned to the nearest promoter region of genes and normalized to the total tag counts for that sample. The mean tag counts of the above mention bins were also calculated. The averaged binned gene expression values were then graphed against mean tag counts for each bin.

### Statistical analysis

Statistical analysis was performed using analysis of variance (ANOVA) followed by post-hoc analyses (StatView 4.02, SAS Institute, Cary, NC). Values are shown as means ± SEM. The null hypothesis was rejected at p < 0.05.

## Abbreviations

ChIP: Chromatin immunoprecipitation; DA: Dopamine; METH: Methamphetamine; H4K5Ac: Histone H4 acetylated at lysine 4; HAT: Histone acetyltransferase; HDAC: Histone deacetylase; IEG: Immediate early gene; IPA: Ingenuity pathway analysis; TSS: Transcriptional start site; Seq: Sequencing.

## Competing interests

The authors declare that they have no competing interests.

## Authors’ contributions

JLC supervised the overall project, designed experiments, and wrote the manuscript.SJ designed, performed and analyzed ChIP experiments, RNA isolation and helped write the manuscript. MTM designed ChIP primer, performed and analyzed ChIP experiments. BL dissected and helped in the drug injections. FSP performed and analyzed RT-PCR experiments. EL performed microarray and ChIP-Seq experiments. SD performed bioinformatics analysis of ChIP-Seq experiments. KGB supervised the overall microarray and ChIP-Seq experiments. CB analyzed the microarray data. All authors read and approved the final manuscript.

## Supplementary Material

Additional file 1 These include Tables S1 to S4. Click here for file
