# Supplementary material for: Genome-wide profiling identifies a subset of methamphetamine (METH)-induced genes associated with METH-induced increased H4K5Ac binding in the rat striatum
Source: BMC Genomics. 2013 Aug 12;14:545. doi: 10.1186/1471-2164-14-545 (PMC3751638; doi:10.1186/1471-2164-14-545)
Supplement: Additional file 1 — These include Tables S1 to S4. [file 1471-2164-14-545-S1.docx]

**Table S1. Schedule of Chronic METH injections and METH Challenge**

|  | Monday | Tuesday | Wednesday | Thursday | Friday |
| --- | --- | --- | --- | --- | --- |
| **Week 1** |  |  |  |  |  |
| 9:00 |  |  |  |  |  |
| 10:00 | 0.5 mg/kg | 1 mg/kg | 1 mg/kg | 1.5 mg/kg |  |
| 11:00 |  |  |  |  |  |
| 12:00 |  |  | 1 mg/kg | 1.5 mg/kg |  |
| 13:00 |  |  |  |  |  |
| 14:00 |  |  | 1 mg/kg | 1.5 mg/kg |  |
| 15:00 |  |  |  |  |  |
| 16:00 | 0.5 mg/kg | 1 mg/kg | 1 mg/kg | 1.5 mg/kg |  |
|  |  |  |  |  |  |
| **Week 2** |  |  |  |  |  |
| 9:00 |  |  |  |  |  |
| 10:00 | 1 mg/kg | 1.5 mg/kg | 2 mg/kg | 2.5 mg/kg |  |
| 11:00 |  |  |  |  |  |
| 12:00 | 1 mg/kg | 1.5 mg/kg | 2 mg/kg | 2.5 mg/kg |  |
| 13:00 |  |  |  |  |  |
| 14:00 | 1 mg/kg | 1.5 mg/kg | 2 mg/kg | 2.5 mg/kg |  |
| 15:00 |  |  |  |  |  |
| 16:00 | 1 mg/kg | 1.5 mg/kg | 2 mg/kg | 2.5 mg/kg |  |
|  |  |  |  |  |  |
| **Week 3** |  | **Test Day** |  |  |  |
| 9:00 |  | 5 mg/kg |  |  |  |
| 10:00 | 3 mg/kg |  |  |  |  |
| 11:00 |  |  |  |  |  |
| 12:00 | 3 mg/kg |  |  |  |  |
| 13:00 |  |  |  |  |  |
| 14:00 | 3 mg/kg |  |  |  |  |
| 15:00 |  |  |  |  |  |
| 16:00 | 3 mg/kg |  |  |  |  |

Initially the rats were divided into two groups, with one group receiving saline and the other group receiving chronic METH according to the schedule described above. They received saline of METH treatment during the first and second weeks as well as Monday of the third week. On Tuesday of the third week, the saline group received either saline (SS) or a single injection of METH (5 mg/kg) (SM) while the chronic METH group was given a single injection of either saline (MS) or METH (5 mg/kg) (MM). All animals were euthanized 2h later.

| **Table S2 List of RT-PCR and ChIP-PCR primers** | |  |
| --- | --- | --- |
|  |  |  |
| **Primer Name** | **Forward Sequence** | **Reverse Sequence** |
| RT-PCR |  |  |
| Arc | CTG GGT GGA GTT CAA GA | CGT CCA CAT ACA GTG TC |
| c-fos | GGG CAA AGT AGA GCA G | CTC TTT CAG TAG ATT GGC A |
| Crem | CCA ACT TAC CAG ATC CGA | TTC TTT AGC AGC TTC CCT |
| Dnajb5 | TTT ACC CTG CAA TGA TGT TAT | GGT TAA TCT GTC TGG GAA T |
| Egr1 | AGC ACT AGA ACA TCA AG | AGA GTT ACA GTC GAG C |
| Egr2 | TAA AGC AAA ACC GAT GT | CTC GAT TCA AGA GAT CAT AA |
| Npas4 | TCT TGG TTG ACC CTG AT | AGG TTG TTG CAA GGT GA |
| Npb | GTT CCG AGT CTC CAG CA | AGA AGA CGT CCG CCT TA |
| Nptx2 | TCA GCC CAC TGT GAA GA | ATT ATG ATA TAT GAT GCA GCC T |
| Nr4a3 | CGT GCA GCA GTT CTA CAA | GAT AGA AAG TCT AAG AAC GAA CA |
| Nts | TTG CAG CCT GAT CAA TAA C | TCC TGA ATT ATC TCC CAG TG |
| Oaz1 | GCT CAC TCC ATT AGC GG | CCA GAC TTC AAA GGA GG |
|  |  |  |
| ChIP-PCR |  |  |
| Arc | CGC CAA ACC CAA TGT GAT | TGC AAC CCT TTC AGC TCT |
| c-fos | TTC TCT GTT CCG CTC ATG ACG T | CTT CTC AGT TGC TAG CTG CAA TCG |
| Egr2 | GGC AGC GAA TCG TTC CGG | TCG GAG TAT TTA TGG GCA GGT |
| Npas4 | GCG ACC AGA TCA ACG CC | CTT GCT TAC CTC CAG CAA AGA A |
| Nr4a3 | CCT GTG TGG AGA ACA GTG AG | ACC TAT GGC CTT AAC CGT C |
|  |  |  |
| For Crem ChIP-PCR, we used a proprietary sequence from Qiagen (GPR1060684(-)03A, Valencia, CA) | | |

| **Table S3. The effects of acute METH on gene expression in control animals** | | |  |
| --- | --- | --- | --- |
| **Symbol** | **Synonyms** | **Definition** | **FC** |
| Dusp14 |  | dual specificity phosphatase 14 | 13.17 |
| Peli1 |  | pellino homolog 1 (Drosophila) | 10.64 |
| Npas4 | Nxf | neuronal PAS domain protein 4 | 9.04 |
| Prodh2 |  | proline dehydrogenase (oxidase) 2 | 6.95 |
| Olr464 |  | olfactory receptor 464 | 6.51 |
| Nr4a3 | NOR-2 | nuclear receptor subfamily 4, group A, member 3 , transcript variant 2 | 6.43 |
| Fos | c-fos | FBJ murine osteosarcoma viral oncogene homolog | 6.18 |
| Nr4a3 | NOR-2 | nuclear receptor subfamily 4, group A, member 3 , transcript variant 1 | 4.71 |
| Egr4 | NGFI-C; Egr4l1 | early growth response 4 | 4.62 |
| Fzd7 |  | frizzled homolog 7 (Drosophila) | 4.22 |
| Pkp2 |  | plakophilin 2 | 3.79 |
| Nptx2 |  | neuronal pentraxin II | 3.77 |
| Inhba |  | inhibin beta-A | 3.71 |
| Dhrs9 | Rdhl | dehydrogenase/reductase (SDR family) member 9 | 3.54 |
| Hcrt | orexin-A | hypocretin | 3.08 |
| Egr2 | Krox20 | early growth response 2 | 3.08 |
| Arc | rg3.1 | activity regulated cytoskeletal-associated protein | 2.88 |
| Junb |  | Jun-B oncogene | 2.36 |
| Taar8c | Ta10 | trace-amine-associated receptor 8c | 2.33 |
| Nts |  | neurotensin | 2.32 |
| Gadd45g |  | growth arrest and DNA-damage-inducible 45 gamma | 2.21 |
| Dnajb5 |  | DnaJ (Hsp40) homolog, subfamily B, member 5 | 2.03 |
| Cebpb | TCF5; LAP; NF-IL6 | CCAAT/enhancer binding protein (C/EBP), beta | 1.99 |
| Tgfb3 |  | transforming growth factor, beta 3 | 1.98 |
| Egr1 | zif-268; Ngf1; Krox-24 | early growth response 1 | 1.89 |
| Baz1a |  | bromodomain adjacent to zinc finger domain, 1A | 1.89 |
| Dusp5 | Cpg21 | dual specificity phosphatase 5 | 1.84 |
| Porf1 |  | preoptic regulatory factor 1 | 1.83 |
| Per1 |  | period homolog 1 (Drosophila) | 1.83 |
| Ptpdc1 |  | protein tyrosine phosphatase domain containing 1 | 1.82 |
| Mchr1 | Slc1; Gpr24 | melanin-concentrating hormone receptor 1 | 1.81 |
| Gpd1 | Gpd3; GPDH | glycerol-3-phosphate dehydrogenase 1 (soluble) | 1.80 |
| Abcc10 |  | ATP-binding cassette, sub-family C (CFTR/MRP), member 10 | 1.78 |
| Crem | CREM-17X; Icer | cAMP responsive element modulator, transcript var 2 | 1.77 |
| Mafk |  | v-maf musculoaponeurotic fibrosarcoma oncogene family, protein K | 1.77 |
| Vgf |  | VGF nerve growth factor inducible | 1.76 |
| Sstr4 | Smstr4 | somatostatin receptor 4 | 1.75 |
| Gadd45b |  | growth arrest and DNA-damage-inducible 45 beta | 1.72 |
| Snf1lk | Sik | SNF1-like kinase | 1.72 |
| Asb1 |  | ankyrin repeat and SOCS box-containing protein 1 | 1.71 |
| Pip3ap |  | phosphatidylinositol-3-phosphatase associated protein | 1.70 |
| Stag3 |  | stromal antigen 3 | -1.85 |
| Gpr149 | Ieda | G protein-coupled receptor 149 | -1.85 |
| Vegfc |  | vascular endothelial growth factor C | -1.90 |
| Jag1 |  | jagged 1 | -1.94 |
| Olr990 |  | olfactory receptor 990 | -4.34 |
| Amy1 | Amy1a | amylase 1, salivary | -4.54 |
| Tshr | TSHRA | thyroid stimulating hormone receptor | -4.85 |
| Dao1 |  | D-amino acid oxidase 1 (Dao1) | -4.94 |
| Plg |  | plasminogen | -5.37 |
| Olr1463 |  | olfactory receptor 1463 | -5.61 |
| Olr462 |  | olfactory receptor 462 | -5.87 |

Gene expression was measured by microarray analysis. Genes were identified as showing changes in expression if they showed greater than + 1.7-fold changes (p < 0.01) in comparison to the control group.

| **Table 4S. Effects of acute METH administration on gene expression in METH-pretreated rats** | | |  |
| --- | --- | --- | --- |
| **Symbol** | **Synonyms** | **Definition** | **FC** |
| Olr577 | Olr577 | olfactory receptor 577 | 6.393442 |
| Npb |  | neuropeptide B | 5.704009 |
| Plagl2 |  | pleiomorphic adenoma gene-like 2 | 4.668333 |
| Cpt1b | M-CPTI; CPT-IB | carnitine palmitoyltransferase 1b, muscle | 3.905844 |
| Ppef2 |  | protein phosphatase, EF hand calcium-binding domain 2 | 3.071544 |
| Gpr143 |  | G protein-coupled receptor 143 | 2.87889 |
| Nr4a3 | NOR-2 | nuclear receptor subfamily 4, group A, member 3 transcript variant 2 | 2.793947 |
| Foxa3 | Hnf3g | forkhead box A3 | 2.600411 |
| Olr734 |  | olfactory receptor 734 | 2.282164 |
| Olfml2a |  | olfactomedin-like 2A | -1.70962 |
| Zfp533 |  | zinc finger protein 533 | -1.73422 |
| Pard3 | Par3 | par-3 (partitioning defective 3) homolog (C. elegans) | -1.73646 |
| Tle4 | Esp2 | transducin-like enhancer of split 4, homolog of Drosophila (Tle4) | -1.75257 |
| Bcar3 |  | breast cancer anti-estrogen resistance 3 | -1.75601 |
| Plag1 |  | pleiomorphic adenoma gene 1 | -1.75873 |
| Rcn1 |  | reticulocalbin 1 | -1.80528 |
| Gpcr12 | Gpr12 | G-protein coupled receptor 12 transcript variant 1 | -1.80588 |
| Arl6 |  | ADP-ribosylation factor-like 6 | -1.86758 |
| Prkcm |  | protein kinase C, mu | -1.88226 |
| Clcn1 | SMCC | chloride channel 1 | -1.89918 |
| Jag1 |  | jagged 1 | -1.93646 |
| Fbxo15 |  | F-box protein 15 | -1.96408 |
| Bmp2 |  | bone morphogenetic protein 2 | -1.99032 |
| Cd44 | CD44A; METAA | CD44 antigen | -2.00766 |
| Rims2 | Nim2; Rim2 | regulating synaptic membrane exocytosis 2 transcript variant 1 | -2.13448 |
| Gpr149 | Ieda | G protein-coupled receptor 149 | -2.14451 |
| Spdya | Gs4 | speedy homolog A (Drosophila) | -2.35315 |
| Plin | PERIA | perilipin | -2.47174 |
| Stag3 |  | stromal antigen 3 | -2.5145 |
| Clca3 |  | chloride channel calcium activated 3 | -3.7743 |
| Cldn22 |  | claudin 22 | -3.88744 |
| Nek1 |  | NIMA (never in mitosis gene a)-related expressed kinase 1 | -4.36424 |
| Olr990 | Olr990 | olfactory receptor 990 | -4.50254 |
| Ctcfl |  | CCCTC-binding factor (zinc finger protein)-like | -5.45068 |
| Sdfr2 |  | stromal cell derived factor receptor 2 | -6.02452 |
| Traf4af1 | C15orf23 | TRAF4 associated factor 1 | -8.82524 |
| Plg |  | plasminogen | -9.93498 |

Gene expression was measured by microarray analysis. Genes were identified as showing changes in expression if they showed greater than + 1.7-fold changes (p < 0.01) in comparison to the control group.
